# The Interaction Between Epigenetic Changes, EMT, and Exosomes in Predicting Metastasis of Colorectal Cancers (CRC)

**DOI:** 10.3389/fonc.2022.879848

**Published:** 2022-05-30

**Authors:** Meiqi Yang, Mingjun Sun, Huijing Zhang

**Affiliations:** Department of Endoscopy, The First Affiliated Hospital of China Medical University, Shenyang, China

**Keywords:** colorectal cancer (CRC), epithelial to mesenchymal transition (EMT), exosomes, DNA methylation, non-coding RNAs (ncRNAs), N^6^-methyladenosine (m^6^A) RNA, metastasis

## Abstract

Worldwide, colorectal cancer (CRC) ranks as the third most common malignancy, and the second most deadly with nearly one million attributable deaths in 2020. Metastatic disease is present in nearly 25% of newly diagnosed CRC, and despite advances in chemotherapy, less than 20% will remain alive at 5 years. Epigenetic change plays a key role in the epithelial-to-mesenchymal transition (EMT), which is a crucial phenotype for metastasis and mainly includes DNA methylation, non-coding RNAs (ncRNAs), and *N* ^6^-methyladenosine (m^6^A) RNA, seemingly valuable biomarkers in CRCs. For ncRNAs, there exists a “molecular sponge effect” between long non-coding RNAs (lncRNAs), circular RNAs (circRNAs), and microRNAs (miRNAs). The detection of exosomes is a novel method in CRC monitoring, especially for predicting metastasis. There is a close relationship between exosomes and EMT in CRCs. This review summarizes the close relationship between epigenetic changes and EMT in CRCs and emphasizes the crucial function of exosomes in regulating the EMT process.

## Introduction

In 2020, colorectal cancers (CRCs) have become significant public health problems worldwide, and their incidence has increased to twice in the top 10 cancers (1–3). Monitoring distant metastatic loci and recurrence *in situ* is the last and most crucial step for cancer treatment. Furthermore, it is the most common cause of concern and anxiety for patients with CRCs. Based on previous studies, the mortality of metastatic CRCs is much higher than that of primary CRCs, especially for liver metastasis. Metastatic cancers severely influence the 5-year survival rate and quality of life of patients (4). Therefore, it is pivotal to predict the metastasis and recurrence of CRCs in order to increase distal survival time and quality of life.

For CRC, epithelial–mesenchymal transition (EMT) is a vital phenotype in its metastasis. EMT is a reversible process that promotes tumor cells exfoliating into circulation and damaging the intercellular skeletal structure. These exfoliated tumor cells could reach distal cites and form metastatic loci. The transcription factors (TFs) *SNAI1*, *SNAI2*, *Zeb1*, *Zeb2*, and *Twist*, called EMT-TFs, and some non-coding RNAs (ncRNAs) can influence the EMT process to regulate the migration and infiltration of tumor cells (5, 6). EMT is a dynamic process that is affected by genetic, epigenetic, and immune environmental factors. N-cadherin, vimentin, matrix metalloproteinases (MMPs), E-cadherins, claudins, epithelial cell adhesion molecules (EpCAMs), and cytokeratins are common biomarkers for the detection of EMT or its reverse process, mesenchymal–epithelial transition (MET). EMT is an essential phenotype in the study of tumor metastasis.

The liquid biopsy (LB) technology is a novel method used to detect the genesis, progress of recurrence, and metastasis in CRC patients. Circulating tumor cells (CTCs), circulating tumor DNAs (ctDNAs), and cell-free RNAs are detectable biomarkers in serum (7). In addition, the detection of exosomes in the urine, cerebrospinal fluid, and saliva is applied widely and clinically. Detecting genetic materials has more perspective as nano-scale biological vesicles that are stable, suitable for storage, and valuable as both biomarkers and therapeutic nano-drugs, exosomes likely have a brighter future than cells (8).

Exosomes are tiny vesicles derived from tumor cells that contain DNA, ncRNAs, epigenetic modulation, and metabolites. Especially in comparison to obtaining tissue biopsies, liquid biopsies, including the relatively noninvasive venipuncture, or sampling other bodily fluids is often safer and more convenient. According to previous studies, exosomes play a pivotal role in predicting cancer metastasis. Comparably, exosome detection is safer and more convenient in malignant cancers, especially for CRCs. LB has shown considerable accuracy in CRC testing. Its sensitivity and specificity can reach up to 60% and 90%, respectively (9, 10). Therefore, this review summarized the relationship between epigenetic changes and EMT in CRCs and emphasized the association between exosomes and EMT.

## Epigenetic Changes and the EMT Process in CRC

The reverse EMT process is characterized by the downregulation of E-cadherin, desmoplakin, claudins, and β-catenin and the upregulation of N-cadherin, vimentin, fibronectin, and Snail1/Snail2 mediate the migration and invasive metastasis of cancer cells (11–13). Whether to promote or inhibit is a question that can be answered using qualified biomarkers in EMT and reflects their influence on tumor migration, infiltration, or more aggressive properties. This process may not be complete; partial EMT is a novel phenomenon that has been recently put forward and proven to be more related to a malignant phenotype. It provides a warning that paying more attention to the regulators during the EMT process is more critical than just the results.

The chromosomal instability (CIN), microsatellite instability (MSI), and the CpG island (CGI) methylator phenotype (CIMP) pathways are three principle pathways involved in the genesis and development of CRCs. CIMP is related to the hypermethylation of promoters or the upstream regulator regions that usually contain CGIs, reported to be related to the detection of CRCs (14, 15). The definition of CIMP depends on the number of methylated loci on these CGIs; these also decide the survival outcomes and long-term prognosis of CRCs (16–18). The CIMP pathway could predict metastasis and recurrence *via* the detection of epigenetic biomarkers, such as DNA methylation, overexpression of ncRNAs, and *N* ^6^-methyladenosine (m^6^A) modification. These epigenetic changes are regulated by genes and can be inherited by the next generation, but they cannot be transmitted into proteins to form phenotypes. Their influence is so strong that they have gained wide attention (19, 20). Several signaling pathways such as the Wnt signaling pathway, Raf/MEK/ERK pathway, and the NF-κB pathway function in CRCs. Especially for the Wnt signaling pathway, canonical Wnt signaling is frequently activated in the tumorigenesis of CRCs, with loss of the adenomatous polyposis coli (APC) genes and the translocation of β-catenin into the nucleus (21–23).

Regarding epigenetic changes, 5-methylcytosine (5mC) methylation for DNA and m^6^A modification for RNA are two epigenetic modifications affecting the transcription and expression of critical genes. In addition, ncRNA is another group that cannot be transcribed, but has high regulatory ability for coding genes. These two mechanisms can undoubtedly regulate the EMT process in CRC metastatic events.

## DNA 5mC Methylation: A Robust Prognostic Biomarker in CRC

DNA methylation occurs when a methyl group is transferred to the carbon-5′ position of the cytosine base (5-methylcytosine) concentrated in CGIs under the catalytic action of DNA methyltransferase enzymes (24). The hypermethylation of 5-mC can regulate not only the transcription elements but also alternative splicing, which can modulate the production of ncRNAs (25–27). The methylation of promoters could weaken the transcription activity into messenger RNA (mRNA) and affect the function of the translated proteins. The intensity of their influence depends on the methylated sites on CGIs, which is differentially sensitive to the distance between the CpG sites among groups (28, 29).

This review focuses on several common and proven effective DNA methylation indicators for CRCs, shown in **Table 1**. The secreted frizzled-related protein (SFRP) family is regarded as an antagonist of the Wnt signaling pathway for its similarity to frizzled (Fz) receptors with bidirectional function (41). SFRP1 and SFRP2 are expressed in stromal myofibroblasts and are downregulated from typical adjacent tumor (normal tissue adjacent to the tumor, NAT) tissues toward the tumor (30, 31, 42, 43). They are diagnostic and prognostic biomarkers and suppress the growth and metastasis of CRC tumor cells. SFRP4 and SFRP5 may also inhibit invasion in CRC, but the underlying mechanisms in regulating the signaling pathways are more complex by comparison (32, 33, 44). Additional studies focusing on the SFRPs family as it relates to CRC should be undertaken to clarify their overall role and potentially derive a novel therapeutic regimen. Different from that of SFRPs, *SDC2* methylation is a carcinogenic biomarker in CTCs tested in patients with CRC (45). Hua et al. conducted a study to prove that the upregulation of SDC2 promotes cell proliferation, migration, and invasion; inhibits apoptosis; and activates and promotes EMT through mitogen-activated protein kinase (MAPK) signaling pathways (34). Apart from that of SFRPs and *SDC2*, the methylation of *TAC1*, *SEPT9*, *HPP1*, *HLTF*, and *NPY* has also been proven to be related to the recurrence and metastasis of CRCs (39). Moreover, the methylation of two factors, hyperplastic polyposis protein 1 (*HPP1*) and helicase-like transcription factor (*HLTF*), has prognostic value for therapy and metastatic location in CRC patients (35, 36, 46, 47). Several DNA methylation have been applied clinically with high sensitivity and specificity. Overall, the panels of methylated *SEPT9* and *SDC2* (48, 49), *SEPT9* and *OSMR* (77.0%) (37), *SEPT9* and *OSMR* (37), *SEPT9* and *TAC1* and *HIC*/*CYCD2*/*VHL* (50), *APC*/*MGMT*/*RASSF2A*/*WIF1* (51), *SYNE1* and *FOXE1* (52), *WIF1*/*NPY*/*PENK* (53), *THBD* and *C9orf50* (54), and the combinations of *FAM123A*/*GLI3*/*PPP1R16B*/*SLIT3*/*TMEM90B* (55) and *MGMT*/*RASSF1A*/*SEPT9* (56) were reported to have diagnostic significance in early-stage CRCs and may be more promising than the use of single markers. The combinations of *BCAT1*/*IKZF1* and *BCAT1*/*IKZF1*/*IRF4* were exploited and were shown to perform better than individual markers (57, 58), with higher sensitivity (73.9%) and specificity (90.1%) and an area under the curve (AUC) of 0.82 (38, 59).

**Table 1:** Potential DNA methylation seen in colorectal cancers (CRCs).

DNA methylation	Expression		Function in CRCs	Reference
SFRP1	Down	Suppressor	Detection, prognosis, and metastasis	(30)
SFRP2	Down	Suppressor	Detection, prognosis, and metastasis	(31)
SFRP4	Up	Suppressor	Detection, prognosis, and metastasis	(32)
SFRP5	Up	Suppressor	Detection, prognosis, and metastasis	(33)
SDC2	Up	Carcinogenic	Detection, prognosis, and metastasis	(34)
HPP1	Up	Carcinogenic	Metastasis and pre-therapeutic outcomes	(35)
HLTF	Up	Carcinogenic	Metastasis and pre-therapeutic outcomes	(36, 37)
SEPT9	Up	Carcinogenic	Metastasis and pre-therapeutic outcomes	(37)
BCAT1	Up	Carcinogenic	Metastasis and pre-therapeutic outcomes	(38)
NPY	Up	Carcinogenic	Metastasis and pre-therapeutic outcomes	(39, 40)

Shown in the table are the usual DNA methylation markers for the prediction of metastasis in CRCs.

Methylated DNA analysis in exosomes has been reported for gastric cancer, prostate cancer, lymphoma, breast cancer, and gingivitis (60). Although an association between DNA methylation in vesicles and CRCs exists, no study on their relationship has been conducted. DNA methylation can regulate the expression of hypoxia-inducible factor (HIF), which can modulate the development and progression of hypoxic CRCs. It is a critical factor in the regulatory EMT process.

## NcRNAs Regulate the EMT Process in CRC

NcRNAs are a class of small RNA fragments or circulars in the non-coding regions. Although an association between DNA methylation in vesicles and CRCs exists, no study on their relationship has been conducted. The ncRNAs in CTCs originate from three main pathways, namely, membrane-bound vesicles, apoptotic bodies, and RNA-binding proteins (RBPs), which have been elucidated in detail in a previous review (61). The ncRNAs can maintain highly stable in the bloodstream with the protection of exosomes or RBPs, such as argonaute-2 (AGO2) high-density lipoprotein (HDL), due to their small size and their ability to escape RNase-mediated degradation (62–66). NcRNAs include a number of RNA types, such as microRNAs (miRNAs), long non-coding RNAs (lncRNAs), and circular RNAs (circRNAs).

MiRNAs were the first discovered ncRNAs that can regulate the functions of genes and proteins. The sequence of miRNAs is short and can form complementary pairs with mRNAs. Most miRNAs are inhibitory and can lead to the degradation of mRNAs, which decreases the expressions of target proteins. Many cytoplasmic miRNAs have been discovered that can regulate expression of N-cadherin, Vimentin, MMPs, E-cadherin, Claudin, and EpCAM, thereby influencing the direction of EMT through molecular regulation in targeted pathways (67–69). Go et al. provided proof that the maternally expressed gene 3 differentially methylated region (MEG3-DMR) can control liver metastasis by regulating miRNAs, such as miR-655-3p, in the 14q32 cluster in CRCs (70, 71). This confirms that circulating miRNAs have an endothelial cell and blood component origin (19, 63, 72). A molecular classification based on epigenetically regulated gene expression profiles was established by Wang et al. to provide a better understanding of the epigenetic mechanisms underlying CRC heterogeneity using The Cancer Genome Atlas (TCGA) dataset and validated across Gene Expression Omnibus (GEO) datasets (73–78).

The upregulation or the downregulation of miRNAs hinges on regulating genes and epigenetic modifications. This variability, the targets and resultant outcomes are exemplified by the following: miR192-2 promoter hypermethylation was shown to modulate tumor metastasis in CRC by regulating the expression of *SOX4* (80). MiR-31 has been reported to increase the sensitivity to 5-fluorouracil (5-FU) at an early stage (81). In the latest study, miR-31 has been proven to increase radiosensitivity in CRC cell lines through targeting *STK40* (80, 82–85).

LncRNAs can promote or suppress the metastasis of tumor cells *via* upregulating or downregulating the process of EMT. LncRNAs have RNA sequences longer than 200 nucleotides that are not translated into proteins (86), so they are more stable than miRNAs (87). Similar to miRNAs, detection of expression or methylation of lncRNAs in CRC has been reported (88). Several lncRNAs, such as *XIST*, *H19*, and *SPRY4-IT1*, are related to EB1, 2/E-cadherin, and vimentin and can be detected from CTCs in plasma (89–91). The lncRNA *H19* can promote CRC metastasis *via* inducing EMT (92). EMT promotes tumor cell growth, proliferation, and metastasis (93). The upregulation of the lncRNA *TUG1* increases the risk of metastasis and further invasion in CRCs *via* activating the Wnt/β-catenin signaling pathway (94). The lncRNA *MALAT1* is a predictive biomarker for metastasis by regulating the epigenetic model (95). Urothelial carcinoma-associated 1 (*UCA1*), which is produced by cancer-associated fibroblasts (CAFs) and released into serum, is associated with the metastasis of CRCs. The upregulation of *UCA1* can change the expressions of the genes in EMT (96). The lncRNA *BANCR* is an oncogenic molecule that enhanced the aggressiveness of CRC metastasis (97). The TGF-β signaling pathway is a critical pathway in regulating cancer-associated expression. The lncRNA activated by TGF-β (*LNCRNA-ATB*) can suppress the expression of E-cadherin in EMT to promote CRC metastasis (98). The lncRNAs *HOTAIR* and *HOXA-AS2* in plasma correlate with the invasion of CRCs *via* EMT (99, 100). The role of lncRNAs in serum is shown in **Table 2**.

**Table 2:** Role of long non-coding RNAs (lncRNAs) and circular RNAs (circRNAs) associated with epithelial–mesenchymal transition (EMT) in colorectal cancers (CRCs).

	Expression	Signaling pathway	Function in CRCs	Reference
Long non-coding RNAs				
*XIST*	Up	ZEB1, 2/E-cadherin, vimentin	Promotes metastasis	(101)
*H19*	Up	ZEB1, 2/E-cadherin, vimentin	Promotes metastasis	(92)
*SPRY4-IT1*	Up	ZEB1, 2/E-cadherin, vimentin	Promotes metastasis	(91)
*TUG1*	Up	Wnt/β-catenin Signaling	Promotes metastasis	(102)
*MALAT1*	Up	Chromatin Remodeling and epigenetic modulation	Promotes metastasis	(103)
*UCA1*	Up	mTOR signaling	Promotes metastasis	(96)
*BANCR*	Up	MAPK/ERK	Promotes metastasis	(104)
*LNCRNA-ATB*	Up	Caspase signaling	Promotes metastasis	(98)
*HOTAIR*	Up	unclear	Promotes metastasis	(100)
*HOXA-AS2*	Up	unclear	Promotes metastasis	(99)
Circular RNAs				
circCSPP1	Up	circCSPP1/miR-193a-5p/COL1A1 axis	Promotes metastasis	(105)
circRNA_0001946	Up	miR-135a-5p/EMT axis	Promotes metastasis	(106)
circ_0026344	Down	Wnt/β-catenin signaling	Inhibits metastasis	(107)
circRNA_0074027	Down	EMT signaling pathway	Inhibits metastasis	(108)
hsa_circRNA_102209	Up	Ras and Rab interactor 1 signaling	Promotes metastasis	(109)
circPTK2	Up	EMT signaling pathway	Promotes metastasis	(110)
hsa_circ_0001178	Up	ZEB1/miR-382/587/616 axis	Promotes metastasis	(111)
hsa_circ_0009361	Down	Wnt/β-catenin signaling	Inhibits metastasis	(112)
circRNA_100876	Up	EMT signaling pathway	Promotes metastasis	(113)
circ-SIRT1	Up	circ-SIRT1/EIF4A3/N-cadherin/vimentin pathway	Promotes metastasis	(114)
circRNA_104916	Down	EMT signaling pathway	Inhibits metastasis	(115)
hsa_circRNA_002144	Up	miR-615-5p/LARP1/mTOR pathway	Promotes metastasis	(116)
circRNA_101951	Up	KIF3A-mediated EMT pathway	Promotes metastasis	(117)
circTBL1XR1	Up	CircTBL1XR1/miR-424 axis	Promotes metastasis	(118)
hsa_circ_0001666	Down	miR-576-5p/PCDH10 axis	Inhibits metastasis	(119)

LncRNAs can regulate CRCs through modulating the EMT process, while circRNAs can regulate CRCs through modulating EMT in tumor tissues.

CircRNAs are novel derivatives from the alternative splicing of lncRNAs that have small ring structures (120, 121). Compared to miRNAs and lncRNAs, circRNAs have shown their fantastic advantage in forecasting the metastasis of CRCs. By analyzing the expression profiles of circRNAs in the six pairs of CRC tissues and adjacent normal tissues, Chen et al. found that about two in three circRNAs were upregulated and one in three circRNAs was downregulated (122). In CRC tissues, the recovery of some circRNAs hints at the possibility of metastasis and recurrence of CRCs. CircRNA-1662 is an oncogenic circRNA that promoted CRC cell invasion and migration through control of EMT, and was reported to be a robust predictor for cancer metastasis (122). CircCSPP1 can promote CRC metastasis by regulating *COL1A1*, which is composed of type I collagen (105). CircRNA_0001946, hsa_circRNA_102209, circPTK2, hsa_circ_0001178, circRNA_100876, circ-SIRT1, hsa_circRNA_002144, circRNA_101951, and circTBL1XR can be overexpressed in CRC to promote metastasis and proliferation (106, 109–111, 113, 114, 116–118). On the other hand, circ_0026344, circRNA-0074027, hsa_circ_0009361, and hsa_circ_0001666 are downregulated in CRC tissues and suppress the metastasis of tumor cells (107, 108, 112, 115, 119). The elevation of circPTK2 in CRC CTCs increases the possibility of tumor metastasis (110). The hsa_circ_0001821 (circ3823) from both CRC tissues and sera may lead to tumor cell infiltration by binding with miR-30c-5p and its target to regulate the expression of *TCF7* (123). The role of circRNAs in CRCs is shown in **Table 2**.

## Interactions Between MiRNAs, LncRNAs, and CircRNAs—The “Sponge Effect”

There are reports that, at times, the outcomes of miRNA transcription and expression is less satisfactory than expected, which hints at the existence of miRNA inhibitors might be present in the tumor microenvironment of CRCs (124). Seitz et al. hypothesized this inhibitor as a “miRNA sponge” that competes with endogenous mRNAs for miRNAs (125). LncRNAs and circRNAs can regulate miRNAs from binding with the target genes by functioning as competing endogenous RNAs (ceRNAs), which can regulate the expressions of genes by competitively integrating with miRNAs (126). The “lncRNA/circRNAs/miRNA/mRNA/protein” pathway is also called the molecular sponge effect (126).

**Figure 1:**
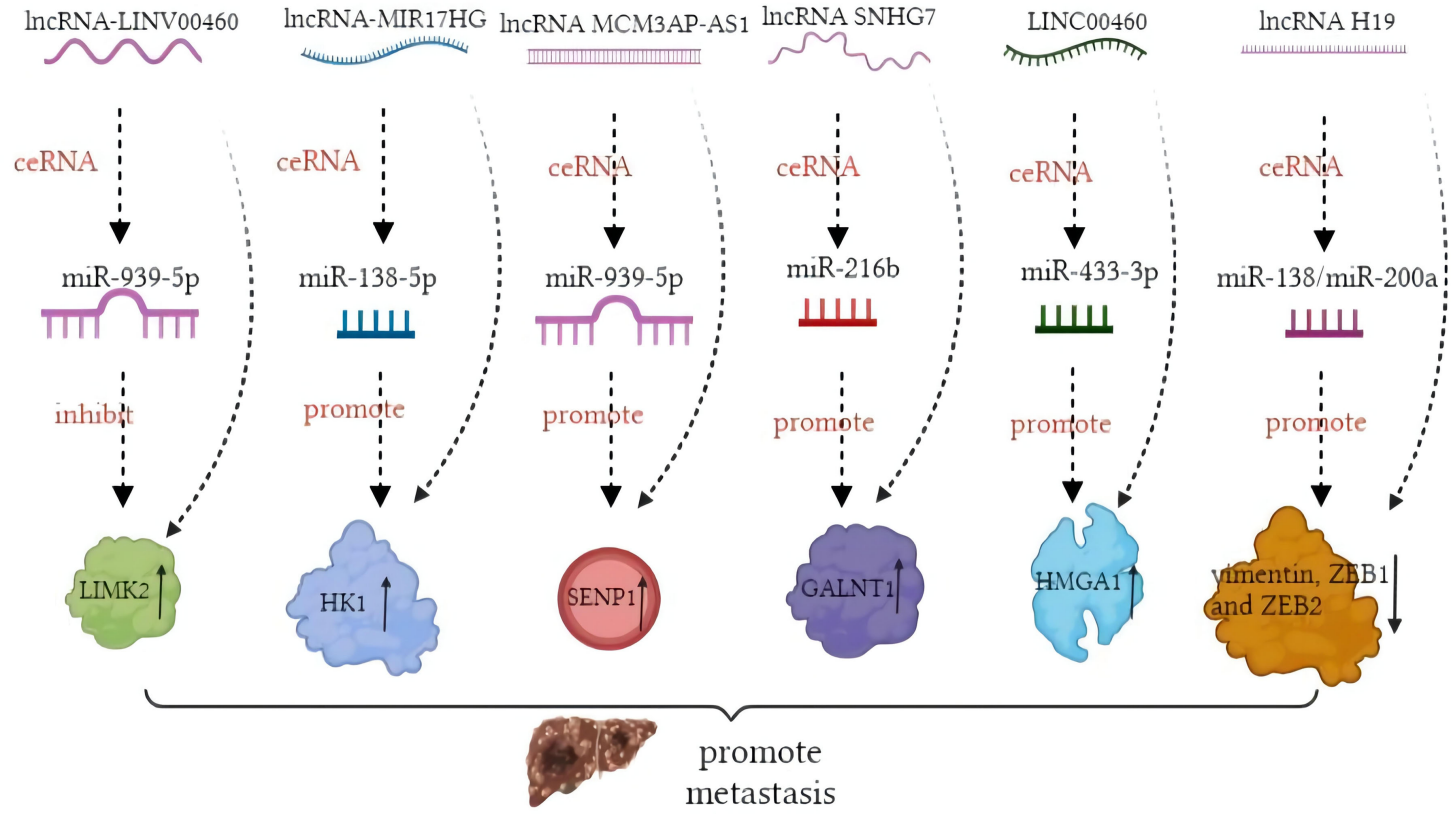
Mechanism of long non-coding RNAs (lncRNAs) acting as competing endogenous RNAs (ceRNAs) in colorectal cancer (CRC) metastasis.

**Figure 2:**
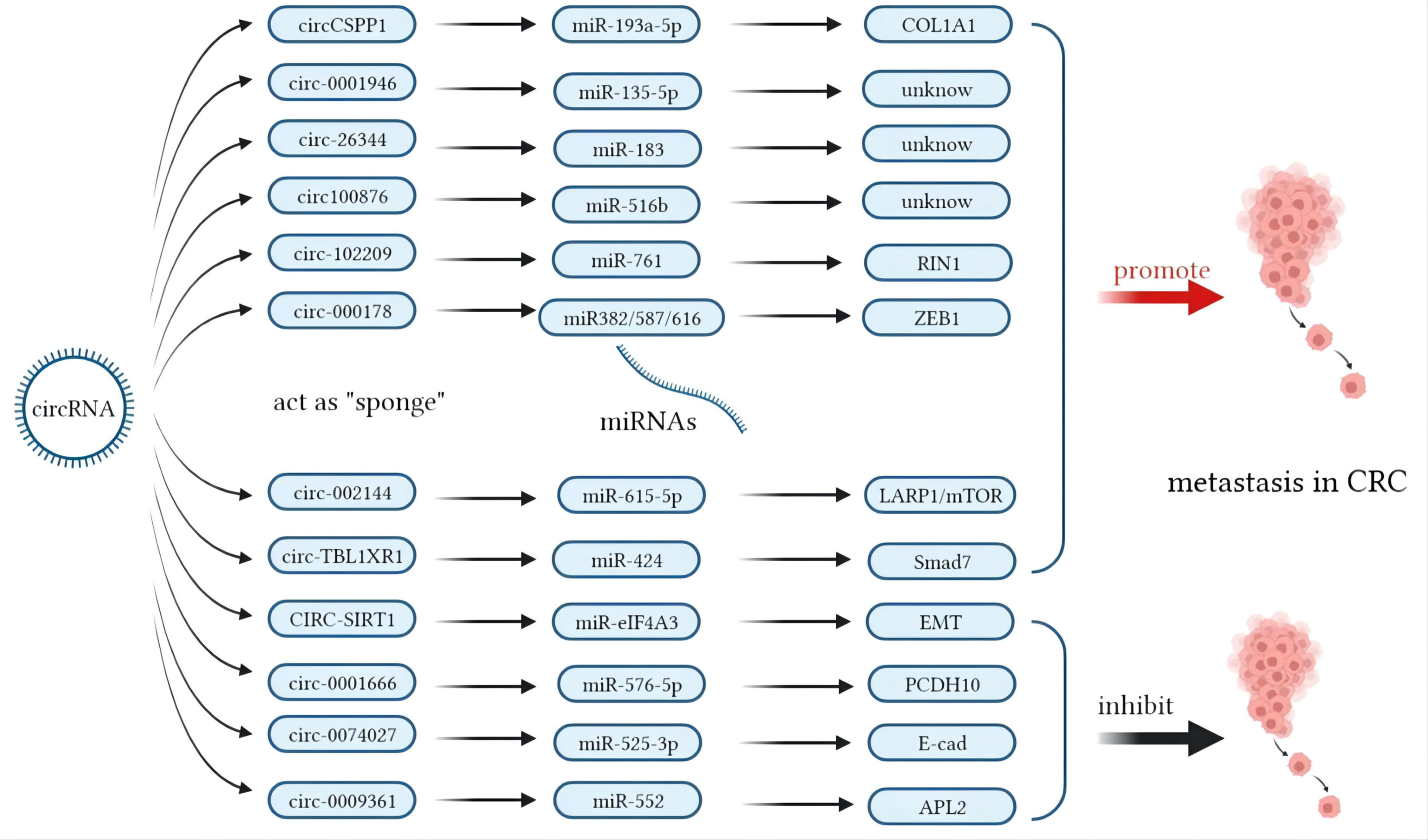
Mechanisms of circular RNAs (circRNAs) acting as competing endogenous RNAs (ceRNAs) in colorectal cancer (CRC) metastasis.

In contrast, miRNA modulation can regulate the expressions of lncRNAs as well. MiR-939-5p is a suppressive miRNA that can inhibit the invasion and metastasis of CRC cells by decreasing the level of *LIMK2*. LncRNA *LINC00460* reduced the inhibition of miR-939-5p on *LIMK2* and bound with miR-939-5p as a ceRNA (127). It is oncogenic through upregulation of annexin to promote the EMT process (128). The glycolysis-related lncRNA *MIR17HG* was found to competitively bind with miR-138-5p to increase glycolysis, which can promote CRC liver metastasis (129). The antisense lncRNA *MCM3AP-AS1* sponged miR-193a-5p through upregulating *SENP1* to promote the progression and migration of CRC (130). The lncRNA *SNHG7* sponges miR-216b to promote the proliferation and liver metastasis of colorectal cancer through upregulating *GALNT1* (131). In the cytoplasm, *LINC00460* is a type of lncRNA that can regulate the expression of high mobility group A1 (*HMGA1*) of m^6^A modification under the catalytic function of methyltransferase 3 (*METTL3*) (132). The earliest found lncRNA, *H19*, functioned as a ceRNA that sequesters miR-138 and miR-200a and leads to the inhibition of vimentin and the protein expressions of *ZEB1* and *ZEB2* (90, 92). The regulatory pathway of lncRNAs on miRNAs is shown in **Figure 1**. Similarly, circRNAs can act as ceRNA sponges of miRNAs (133). Most circRNAs positively affect target genes and promote the metastasis of CRCs. A few circRNAs act as suppressors of tumor invasion and promotion. Circ_0026344, hsa_circ_0009361, and hsa_circ_0001666 are negative regulators of tumor proliferation and inhibit metastasis. The sponge mechanism of circRNAs on miRNAs is shown in **Figure 2**.

## The Role of M6A Methylation of RNAs in EMT

M6A methylation is a novel and critical component of RNA modification related to the expressions of ncRNAs in epigenetics (134). M6A in RNA post-transcriptionally regulates mRNAs by affecting the splicing, export, stability, and translation of transcripts (135–138). Its methylation regulates miRNA synthesis, processing, and maturation, which are crucial in tumorigenesis and cancer progression (139). It has been reported that the m^6^A modification could alter its local RNA structure at the terminal loop region of primary miRNAs (pri-miRNAs) to promote their processing through nuclear transcripts and alternative splicing by modulating RALY binding (140–142). It is promising that m^6^A methylation can predict metastasis and the prognosis of therapy (143). The upregulation of *HMGA1* of m^6^A modification in CRC cells enhances the probability of metastasis (132).

In total, 11 readers, 7 writers, and 2 erasers have already been found. The term “writers” often denotes methyltransferase as the beginning of m^6^A methylation, and different methyltransferases form a complex to gain more powerful catalytic ability. The (143)methyltransferase complex (MTC) comprises the m^6^A/METTL complex (MAC) and the m^6^A/METTL-associated complex (MACOM). MAC consists of *METTL3* and *METTL14*, which can form stable heterodimers (144). Besides *METTL3* and *METTL14*, *WTAP* is another writer that can participate in the formation of complex to regulate m^6^A. NcRNAs can affect the process of m^6^A by regulating the function of the “writer” complex. For example, *METTL3* is the first discovered RNA methylation-related mRNA with a catalytic subunit and is upregulated in metastasis to promote cell migration and invasion in CRCs by regulating miR-1246 (145). Contrarily, *METTL14* is regulated to suppress cell proliferation, invasion, and migration in CRCs *via* miR-375 (146–149). Furthermore, there exists mutual action between m6A modulation and ncRNAs. Prior studies demonstrated that knockdown of METTL3 downregulated the expression levels of miR-483, miR-676, miR-877 and circ1662-YAP1-SMAD3 axis in regulating CRC invasion (122, 142). The m^6^A modification of circRNA_103783 (also called circNSUN2) could increase export into the cytoplasm in the circNSUN2/*IGF2BP2*/*HMGA2* ternary complex (150). In addition, the high expression of circNSUN2 predicts a more aggressive characteristic of CRC; thus, m^6^A modification enhances the metastasis risk of CRC cells (150).

Beyond the methyltransferases, the “readers” that can recognize m^6^A modulation sites, such as *YTHDF1*, *IGF2BP1*, *IGF2BP3*, and *EIF3B* (151), are also under the influence of ncRNAs. The tumor-suppressive miR-1266 promoted the occurrence and progression of CRCs by directly targeting *FTO* (152). Moreover, the expression of the lncRNA *GAS5* can reversely regulate *YAP1 via* m^6^A (153). Surprisingly, the levels of m^6^A can positively increase with the increased concentrations of circRNAs (154). The upregulation of the lncRNA *RP11* by m^6^A methylation can accelerate the spread of CRC cells through the upregulation of *ZEB1* (155). Interestingly, it has been proven that m^6^A circRNAs are different from m^6^A mRNA sites. This indicates that disturbance in the m^6^A modification of mRNA is not equivalent to that of m^6^A circRNAs. Therefore, to abolish the effects of m^6^A modification, then the m^6^A of circRNAs must be considered as well (123, 154). Therefore, to abolish the effects of m^6^A modification, then the m6A of circRNAs must be considered as well. NcRNAs can regulate the critical enzymes as the downstream target molecules or combined partners. Feedback of the essential enzymes affects the m^6^A modulation of ncRNAs, and this process can change the cancer-related phenotype and lead to metastasis (155).

## The Exosomes and EMT in CRC

What is an exosome? Exosomes are microvesicles with diameters from 30 to 150 nm. They are derived from normal intestinal cells, CRC cells, and other cancer-related stromal cells (156). Exosomes have various physiological functions and participate in multiple cancer-related signaling pathways. Exosomes derived from CRCs cells are associated with a metastatic phenotype in CRCs, such as migration, infiltration, and EMT. For exosomes, surface antigens act as biomarkers that contribute to recognition, and their components determine the tasks exosomes carry out. Exosomes are secreted not only from cancer cells but also from normal intestinal cells or other tumor-related cells. Hepatocytes and fibroblasts can also produce exosomes to enter into the blood, urine, or saliva (157, 158). NcRNAs excreted from exosomes play an important role in regulating the signaling pathways in CRC metastasis (159). Studies that excluded the interference of miRNAs and lncRNAs in cells *via* the addition of anti-miRNAs and exogenous exosomes containing miRNAs confirmed that ncRNAs negatively regulated the downstream target proteins or mRNA molecules to promote tumor metastasis *via* EMT (160–162). In contrast, EMT cancer cells can promote exosome secretion *via* increasing the permeability of vascular epithelial layers, and miR-27b-3p can regulate this process through the STAT3 pathway (163). There is a mutual regulation between exosomes and the EMT process, but most studies have focused on the influence of exosome transportation in EMT, as shown in **Table 3**. While the ncRNAs in exosomes can negatively regulate the target genes and promote tumor metastasis, this also hints that they may be brilliant therapeutic targets for the prevention of metastasis. Besides CRC cells, tumor-associated macrophages (TAMs) comprise another class involved in the regulation of exosome and regulate the EMT process in inflammatory or immune diseases (164). The effects being positive or negative depend on the inflammatory cells and cytokines that TAMs secrete. The miRNAs or lncRNAs wrapped in vesicles could affect the M1 or M2 polarization of TAMs (172).

**Table 3:** Functions of exosomes in colorectal cancer (CRC) metastasis *via* regulating the epithelial–mesenchymal transition (EMT) process.

Author	Source of exosomes	Components in exosomes	Target molecules	Regulated phenotype	Outcomes	Reference
**J.L. Hu**	Cancer-associated fibroblasts (CAFs)	miR-92a-3p	*FBXW7* and *MOAP1*	EMT	Metastasis	(160)
**T. Liu**	normal intestinal FHC cells	miR-128-3p	*BMI1* and *MRP5*	EMT	Metastasis and oxaliplatin resistance	(161)
**Z. Liang**	CRC cells	LncRNA RPPH1	*TUBB3* and macrophage M2 polarization	EMT	Metastasis	(162)
**H. Xu**	hepatocyte	miR-203a-3p	Src	EMT	Metastasis	(158)
**D. Wang**	CRC cells	miR-25-3p, miR-130b-3p, and miR-425-5p	PTEN through PI3K/Akt signaling pathway/macrophage M2 polarization	EMT	Metastasis	(164)
**R. Rezaei**	CRC cells	miRNA-375-3p	β-catenin, vimentin, *ZEB1*, and *SNAIL*	EMT	Metastasis	(165)
**X. Zhang**	CRC cells	miR-1255b-5p	Human telomerase reverse transcriptase (hTERT) and *BRG1*	EMT	Metastasis	(166)
**T. Li**	Mesenchymal stem cells	microRNA-3940-5p	Integrin α6 and TGF-β1	EMT	Metastasis	(157)
**H. Liu**	CRC cells	miR-106b-3p	DLC-1	EMT	Metastasis	(167)
**L. Zhou**	Cancer-associated fibroblasts (CAFs)	LncRNA LINC00659	miR-342-3p/*ANXA2* axis	Proliferation, invasion, migration, and EMT	Metastasis	(168)
**Z. Xiao**	CRC cells	miR-1915-3p	*PFKFB3* and *USP2*	EMT	Metastasis	(169)
**F. Mannavola**	CRC cells	miR-106b-3p	Deleted in liver cancer 1 (*DLC1*)	EMT	Metastasis	(170)
**Y. Xu**	CRC cells	circRNA FBXW7	miR-18b-5p	EMT and oxaliplatin resistance	Metastasis	(171)

## Conclusion

Cancer metastasis has always been a hot topic for scientists and clinicians, and discovering the risk factors and suitable therapeutic targets is a critical part of preventing metastasis. Epigenetic change is a popular context associated with the progression and metastasis of CRCs due to its variability and vulnerability. NcRNAs plays a crucial role in regulating epithelial tissues and the mesenchymal components. The dynamic process determines the tumor metastasis tendency. In this regulation, the sponge effect between lncRNAs/circRNAs/miRNAs/mRNA is critical. In addition, epigenetic modification is a crucial link. The 5mC methylation of DNA and the m^6^A methylation of RNA in CRCs could enhance the invasive and migration ability to promote metastasis. The mechanisms of the mutual effect between methylation and ncRNAs may provide a novel direction for future studies.

The EMT is a vital regulator in metastasis, while the mechanism and influence factor of EMT is still unclear. As mentioned before, these epigenetic changes are not independent but interacting. The methylation of promoters can regulate the process of alternative splicing and production of ncRNAs. The review interprets a crosstalk interaction in epigenetic changes from DNA methylation, ncRNAs, and m6A methylation. The function of epigenetic changes is powerful and complex in regulating mechanisms in CRC metastasis. At the same time, EMT is a cross point that the DNA methylation, m6A methylation, and ncRNAs can all affect CRC metastasis. They can upregulate or downregulate the biomarkers in EMT such as N-cadherin, Vimentin, MMPs, E-cadherin, Claudin, and EpCAM to control the progress of the epithelial transition.

Another novelty of the review is that the exosomes can regulate EMT as a carrier for transporting ncRNAs. The function of exosomes has gained widespread attention in cancers. The exosomes have been proved to be related to cancers detection. This review summarizes the effect of exosomes in CRC metastasis related to EMT. That indicates that exosomes are a vital factor in the tumor micro-environment. The tumor cells or other stromal cells can secret exosomes to regulate EMT. And this review indicates that DNA methylation, ncRNAs, m6A methylation, exosomes, and EMT can all act as potential therapeutic targets in CRC metastasis. The associated relationship and mutual interaction among them are elucidated thoroughly in this review. While prior studies have been published elucidating the mechanisms of exosomes and EMT, the field remains in its relative infancy, with the potential to discover important new therapeutic targets for CRC.

The detection of exosomes in LB is a novel method to predict metastasis in CRCs. The variety of stromal cells can produce exosomes to participate in the progression, proliferation, migration, and EMT in CRCs. Epigenetic changes, ncRNAs, and exosomes are not independent of each other; on the contrary, there exists an intrinsic interrelation between them. Different from previous studies, this review elaborated on the potential links between epigenetic changes, exosomes, and EMT in CRCs regarding metastasis. It provided novel sights for the study of probable mechanisms of metastasis in CRCs. There have already been several studies on the mechanisms of exosomes and EMT, but more studies are needed to discover new therapeutic targets for CRCs.

## Author Contributions

MY and HZ designed the study design and wrote the main manuscript. MS contributed to the final proof. All authors reviewed the manuscript. All authors contributed to the article and approved the submitted version.

## Conflict of Interest

The authors declare that the research was conducted in the absence of any commercial or financial relationships that could be construed as a potential conflict of interest.

## Publisher’s Note

All claims expressed in this article are solely those of the authors and do not necessarily represent those of their affiliated organizations, or those of the publisher, the editors and the reviewers. Any product that may be evaluated in this article, or claim that may be made by its manufacturer, is not guaranteed or endorsed by the publisher.
